# Primary Syphilis Presenting As a Chronic Lip Ulcer

**DOI:** 10.7759/cureus.7086

**Published:** 2020-02-24

**Authors:** Claire Porterfield, David Brodell, Lindsey Dolohanty, Glynis Scott

**Affiliations:** 1 School of Medicine, University of Rochester, Rochester, USA; 2 Dermatology, University of Rochester, Rochester, USA; 3 Dermatopathology, University of Rochester, Rochester, USA

**Keywords:** chronic ulcer, primary syphilis, syphilis, chancre, histology, skin, cutaneous, biopsy, oral syphilis, treponema pallidum

## Abstract

Syphilis is usually a sexually transmitted infection caused by the spirochete Treponema pallidum. Primary syphilis classically presents as a painless, ulcerated lesion on the genitals. However, the primary lesion is not restricted to this site and appears wherever the spirochete enters through the skin. The symptomatology and appearance of the primary lesion can also vary.

We present a case of a 59-year-old man with a primary syphilitic chancre of the lower lip. The patient was referred to the dermatology clinic by their primary care provider after the ulceration failed to heal with antibiotic therapy. A biopsy of the lesion was taken at this time; the diagnosis of syphilis was then made by histologic examination and immunohistochemical staining. Subsequent serologic tests were also positive. Upon prompting, the patient did report a history of sexually transmitted disease but not of syphilis specifically. The patient was treated with penicillin, and there was clinical improvement of the lesion at the follow-up visit.

## Introduction

Primary syphilis classically presents as a painless, ulcerated lesion on the genitals. However, it can present in other locations, such as the lips, and with variable appearance, such as painful herpetic-like lesions or vascular pyogenic granuloma-like lesions [1,2]. The diagnosis can be made by serologic testing or by histologic examination. Once the diagnosis of syphilis has been established, treatment is usually straightforward; penicillin is the standard treatment and doxycycline is an acceptable alternative in most cases [3].

## Case presentation

Initial history and presentation

A 59-year-old male presented to his primary care physician with a two-week history of an excoriated “pimple” on his left chin. The patient denied any previous similar lesions. The patient had a history of asthma, hypertension, depression, arthritis, and chronic back pain. He vaguely recalled a resolved sexually transmitted infection many years before. His only medications were losartan, albuterol, and ibuprofen.

**Figure 1:**
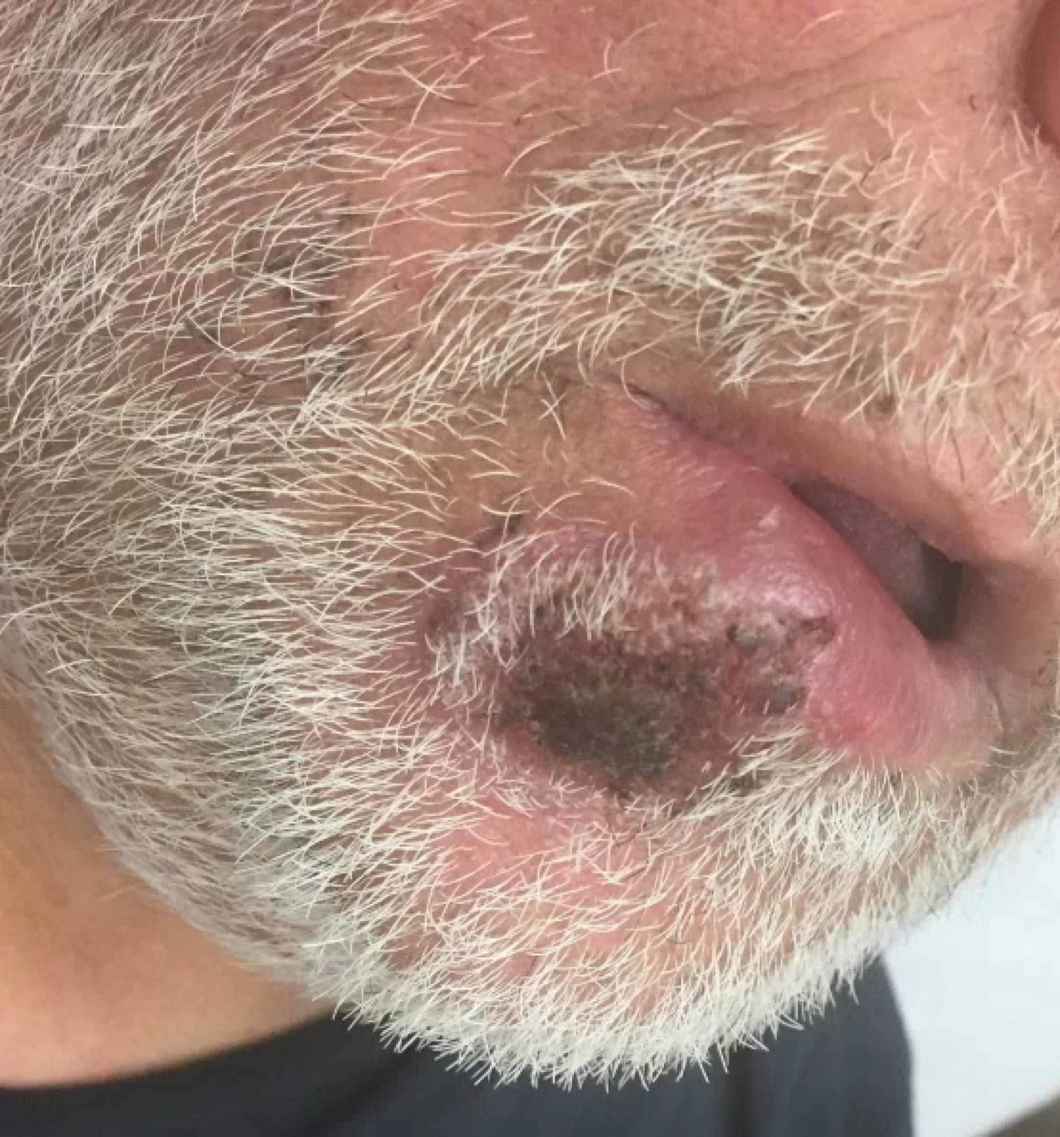
Syphilitic chancre presenting as hemorrhagic, crusted plaque on the right cutaneous lip with associated edema.

Physical examination revealed a tender 2 × 3 cm, edematous, hemorrhagic, crusted plaque on the left lower lip that crossed the vermillion border (Figure 1). No purulence was present. The oral mucosa was normal, and there was no lymphadenopathy. The patient was afebrile.

The lesion did not respond to a course of oral clindamycin prescribed by his primary care provider before seeing dermatology; subsequent incision and drainage revealed a bloody discharge without purulence or signs of gross infection.

Further work-up

**Figure 2:**
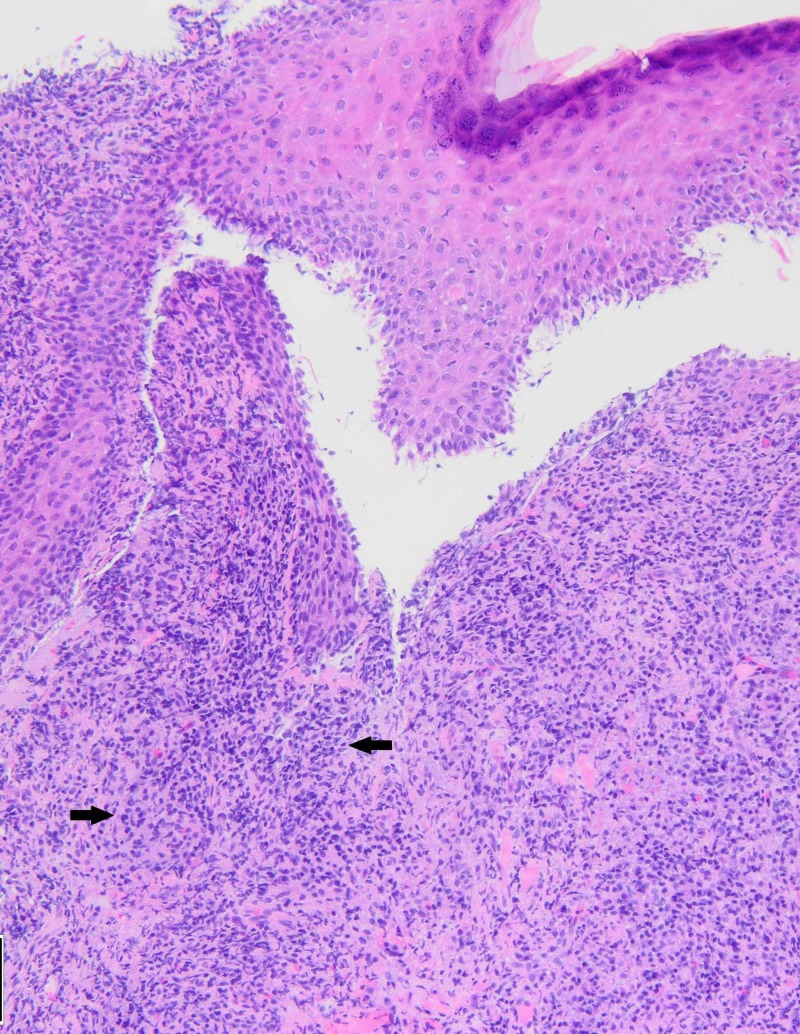
Hematoxylin and eosin staining of a skin biopsy at 10x magnification. The skin is oriented with the surface at the top of the image. There is a diffuse, dense inflammatory cell infiltrate that fills the upper dermis. Within this inflammatory infiltrate are abundant plasma cells (arrows), which are discernible by their hyperchromatic (dark purple) nuclei.

**Figure 3:**
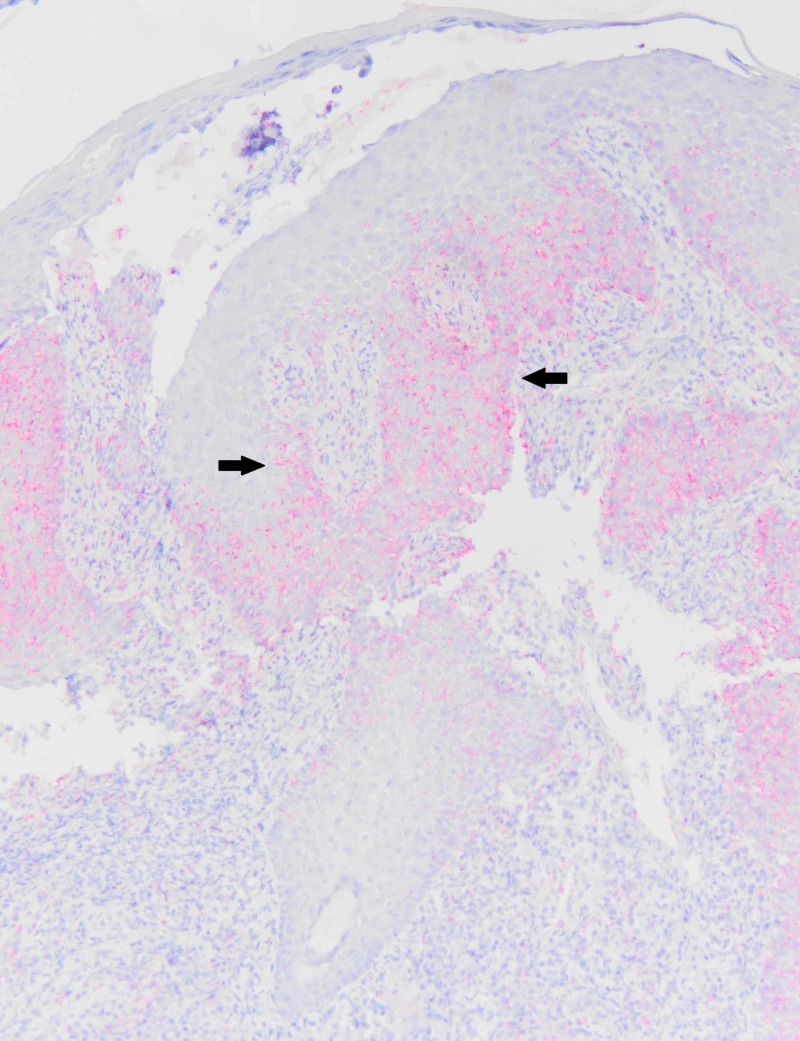
Immunohistochemical stain for Treponema pallidum (10x magnification). The skin is oriented with the surface at the top of the image. Within the epidermis, the stain highlights thin Treponema pallidum organisms in pink (arrows).

On referral to a dermatology clinic, a punch biopsy was performed given concern for squamous cell carcinoma or deep fungal infection. The biopsy revealed a lichenoid infiltrate associated with numerous plasma cells (Figure 2). The presence of plasma cells prompted immunohistochemical staining for Treponema pallidum, which revealed numerous organisms (Figure 3). A diagnosis of primary syphilis was made.

Rapid plasma reagin (RPR) serologic testing and the Treponema pallidum particle agglutination assay were positive, consistent with this diagnosis. Additional screening for human immunodeficiency virus (HIV), *C*hlamydia trachomatis, Neisseria gonorrhoeae, and hepatitis B and C infection was negative.

## Discussion

Current review of syphilis in the United States

The incidence of syphilis in the United States has been steadily rising since 2001, with 25,133 cases diagnosed in 2016. Initially, this increase was primarily among men, especially men who have sex with men. However, more recently, increases have been seen in both the male and female population [4].

Syphilis enters the human body through microabrasions in the skin. Approximately 21 days after exposure, a primary syphilitic chancre appears. It is frequently a painless papule with a propensity to ulcerate. Chancres are not associated with an exudate, but regional lymphadenopathy is often present [5]. The great majority of chancres present as genital lesions; however, they can present at other sites, as in this case, with 10% appearing on the anus, 4% orally, and 1% elsewhere [2].

Multiple primary chancres can occur, especially in patients co-infected with HIV. The chancre will usually resolve without treatment in a matter of weeks [6]. Primary lesions are highly infectious with a transmission rate of 30%; in contrast, latent lesions have low infectious potential [5]. Oral sex plays an important role in the transmission of syphilis, with around 14% of patients reporting this as their only risk factor for the disease [7]. When the lesions do present on the genitals, they often do not resemble the classic ulcerated lesion seen in our patient. They can be painful, resembling genital herpes, nodular, or lichen planus-like lesions [1].

Treatment

The treatment of syphilis is determined by the stage of the disease: early manifestations are treated with penicillin, 2.4 million units once; treatment for late latent or tertiary syphilis is treated weekly and neurosyphilis is treated hourly. Doxycycline can be used as an alternative for penicillin-allergic patients, although this has not been proven efficacious in neurosyphilis or syphilis in pregnant women [3]. 

The disease becomes more difficult to treat over time; however, treatment early in the patient's disease course (in the primary, secondary, or early latent stages) is usually curative, resulting in disease progression only 10% of the time [8]. Treatment in the early stages can precipitate a Jarisch-Herxheimer reaction, manifesting as fever, headache, and hypotension, which should not be mistaken for a penicillin allergy. Patients should be monitored serologically for decline in RPR titers at 6 and 12 months. The majority of patients treated in the early stages of disease will serorevert by 36 months [3].

**Figure 4:**
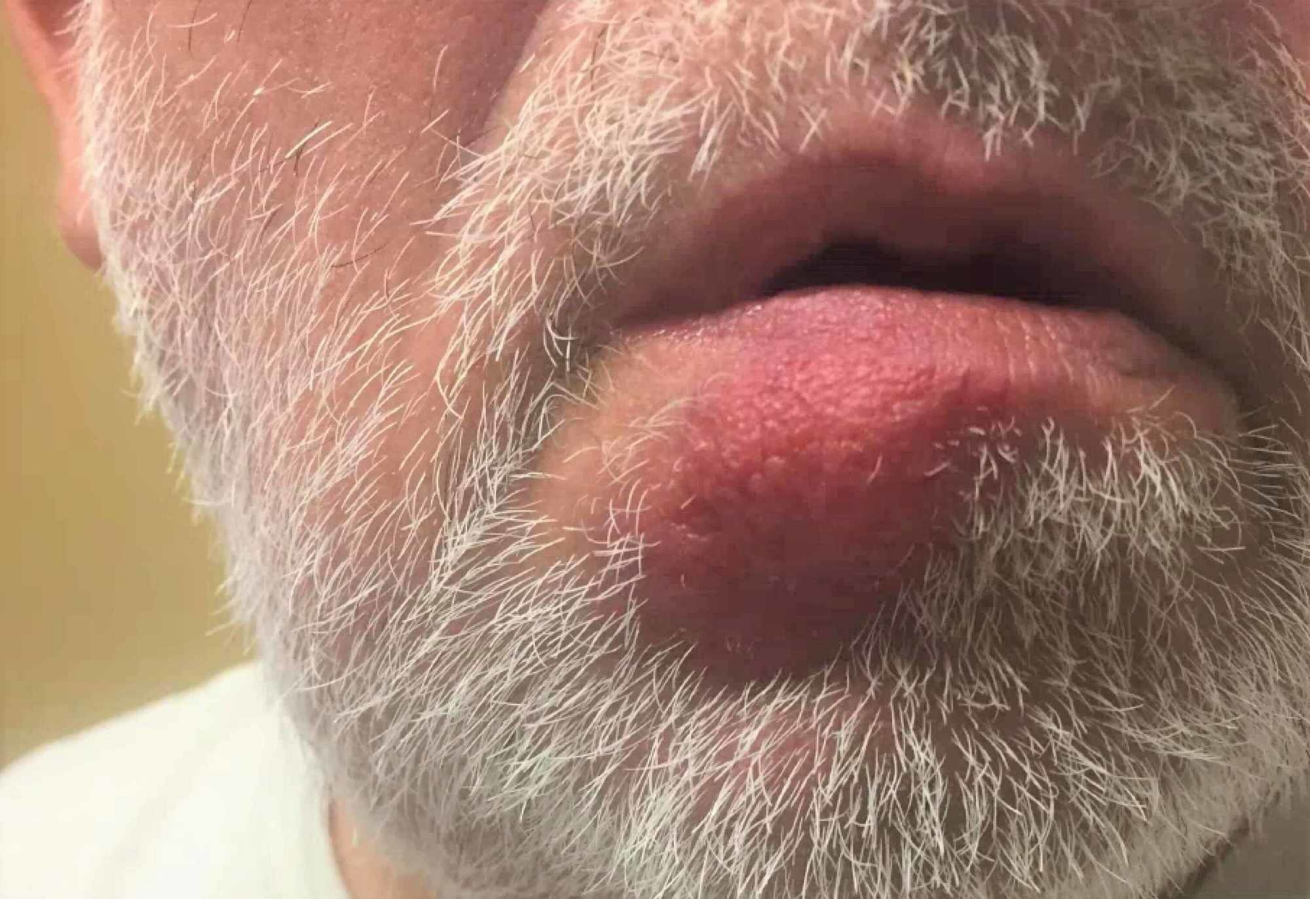
Resolving syphilitic chancre of the left cutaneous lip, four weeks after treatment.

Our patient was treated with a single dose of benzathine penicillin, 2.4 million units. Four weeks after treatment, his primary chancre was dramatically improved (Figure 4).

## Conclusions

For any non-healing ulcer, the differential diagnosis is broad, including chancroid, herpes simplex, tuberculous chancre, deep fungal infection, cellulitis, malignancy, traumatic ulcer, and aphthous stomatitis. Our patient highlights the need for a high index of suspicion for syphilitic chancres presenting in non-genital locations. The early diagnosis of primary syphilis is important given its high incidence of transmission and the serious consequences associated with untreated disease.
